# Dysfunction of Autonomic Nervous System in Childhood Obesity: A Cross-Sectional Study

**DOI:** 10.1371/journal.pone.0054546

**Published:** 2013-01-24

**Authors:** Petra Baum, David Petroff, Joseph Classen, Wieland Kiess, Susann Blüher

**Affiliations:** 1 Department of Neurology, University of Leipzig, Leipzig, Germany; 2 Clinical Trial Centre, University of Leipzig, Leipzig, Germany; 3 Integrated Research and Treatment Center (IFB) Adiposity Diseases, University of Leipzig, Leipzig, Germany; 4 Department of Women and Child Health, University Hospital for Children and Adolescents, Leipzig, Germany; Scientific Directorate, Bambino Hospital, Italy

## Abstract

**Objective:**

To assess the distribution of autonomic nervous system (ANS) dysfunction in overweight and obese children.

**Methods:**

Parasympathetic and sympathetic ANS function was assessed in children and adolescents with no evidence of impaired glucose metabolism by analysis of heart rate variability (low frequency power ln(LF), high frequency power, ln(HF); ln(LF/HF) ratio, ratio of longest RR interval during expiration to shortest interval during inspiration (E/I ratio), root mean square of successive differences (RMSSD); sympathetic skin response (SSR); and quantitative pupillography (pupil diameter in darkness, light reflex amplitude, latency, constriction velocity, re-dilation velocity). The relationship of each ANS variable to the standard deviation score of body mass index (BMI-SDS) was assessed in a linear model considering age, gender and pubertal stage as co-variates and employing an F-statistic to compare the fit of nested models. Group comparisons between normal weight and obese children as well as an analysis of dependence on insulin resistance (as indexed by the Homeostasis Model Assessment of Insulin Resistance, HOMA-IR) were performed for parameters shown to correlate with BMI-SDS. Statistical significance was set at 5%.

**Results:**

Measurements were performed in 149 individuals (mean age 12.0 y; 90 obese 45 boys; 59 normal weight, 34 boys). E/I ratio (p = 0.003), ln(HF) (p = 0.03), pupil diameter in darkness (p = 0.01) were negatively correlated with BMI-SDS, whereas ln(LF/HF) was positively correlated (p = 0.05). Early re-dilation velocity was in trend negatively correlated to BMI-SDS (p = 0.08). None of the parameters that depended significantly on BMI-SDS was found to be significantly correlated with HOMA-IR.

**Conclusion:**

These findings demonstrate extended ANS dysfunction in obese children and adolescents, affecting several organ systems. Both parasympathetic activity and sympathetic activity are reduced. The conspicuous pattern of ANS dysfunction raises the possibility that obesity may give rise to dysfunction of the peripheral autonomic nerves resembling that observed in normal-weight diabetic children and adolescents.

## Introduction

Obesity has reached global epidemic proportions [1] with prevalence remaining high, despite some evidence of stabilisation [2]. Childhood obesity is associated with a number of metabolic, cardiovascular and other disturbances, which include lipid abnormalities, altered glucose metabolism and type 2 diabetes, pulmonary disorders including obstructive sleep apnoea and reactive airway disease, a pro-inflammatory state and coagulation abnormalities [3]. Because dysfunction of the autonomic nervous system (ANS) may contribute to development or stabilization of obesity and is associated with cardiovascular mortality [4], the study of ANS function in obesity is of considerable clinical interest. Most previous studies of ANS function in obese children have been limited to investigations of cardiac autonomic function through analysis of heart rate variability. These studies have generally revealed a decrease in parasympathetic activity. It remains unclear, however, whether abnormalities of ANS can be found in the sympathetic nervous system, and outside of the cardiac autonomic nerve function in overweight and obese children and adolescents.

Methods to assess autonomic nervous system function outside the cardiac system include sympathetic skin response (SSR) and quantitative pupillography. SSR is a simple, reproducible method to measure the polysynaptic reflex loop which comprises diverse afferents, a common efferent pathway through the spinal cord, pre- and postganglionic sympathetic fibres, and perspiratory glands as effectors. Hence, SSR may be affected by pathology of both the peripheral and central sudomotor nervous systems [5], [6]. In obese adults, SSR is not altered [7]. However, SSR has, to the best of our knowledge, not been assessed previously in overweight or obese children.

Autonomic innervation of the pupil is controlled by both the sympathetic and the parasympathetic nervous systems [8]. Pupillary reaction may be quantified via direct pupillography by measuring the pupil diameter in the dark (PDD) and following exposure to a light stimulus. Pupil size and re-dilation velocities (early and late) are thought to reflect sympathetic pupillary modulation while light reflex amplitude, latency and constriction velocity are controlled by parasympathetic activity [8]. PDD and amplitude, latency of pupillary response and velocity of pupillary constriction and of re-dilation following light stimulation are impaired in diabetic patients and may represent the earliest manifestations of peripheral autonomic neuropathy [9]–[11]. In diabetic adolescents, pupillary dilation and the PDD are significantly decreased compared to healthy peers, and the degree of the impairment correlates with the duration of the disease [12], [13]. However, if and how pupillary function is influenced by obesity has not previously been studied.

The aim of this cross-sectional study was to investigate whether abnormally increased body weight in children and adolescents, as indexed by the standard deviation score of body mass index (BMI-SDS [14], [15], is associated with abnormalities of sympathetic and parasympathetic ANS function. Because impaired glucose tolerance and type 2 diabetes mellitus as well as some medication used to treat co-morbidity are well known to impair ANS function [16], [17] these potential confounders were carefully excluded.

## Subjects and Methods

### Subjects

Normal weight or overweight/obese children or adolescents were eligible for the study conditional upon normal glucose metabolism as tested in an oral glucose tolerance test (overweight/obese participants).

*Inclusion criteria* were as follows: age 7–18 years, body mass index (BMI) between 10^th^ and 90^th^ percentile (normal weight) or 90^th^–97^th^ percentile (overweight) or >97^th^ percentile (obese), respectively, according to German reference values [14]. Overweight and obese individuals are from here on referred to as the “obese” group.

*Exclusion criteria* were: neuromuscular disorder, systemic neoplasia or impaired glucose tolerance (serum glucose levels >7.8 mmol/l, measured 120 min after an oral glucose tolerance test (OGTT)) [18]. In addition, candidates with monogenetic causes of obesity were also excluded. To date, more than 50 genetically based syndromes are associated with obesity, which are in most cases accompanied by more or less typical clinical features. Frequent disorders of syndromal obesity include Prader-(Labhart)-Willi syndrome, Bardet-Biedl syndrome, Alström syndrome, Cohen syndrome, Carpenter syndrome, fragile X syndrome, and others [19].

Recruitment was performed via the obesity outpatient clinic of the Children’s Hospital, University of Leipzig (for adipose candidates) and via local newsletter postings (for normal weight children) between 2010 and 2011.

Written informed consent was obtained by all participants and their caregivers/parents of our study. The study protocol was approved by the Ethics Committee of the Faculty of Medicine of the University of Leipzig.

Detailed baseline characteristics of both study groups are given in Table 1.

**Table 1:** Characteristics of study cohorts.

	Ov/Ob (>90^th^ percentile)		Normal weight (10^th^ –90^th^ percentile)		p-value
	male	female	male	Female	normal weight vs. ov/ob
	n = 45	n = 45	n = 34	n = 25	
**Age** (years)	12.2±2.3[7.9–17.9]	11.9±2.7[7.4–17.5]	12.4±2.6[8.0–17.3]	11.5±2.6[7.3–16.5]	0.8
**Weight** (kg)	71.6±16.4[44.4–117.2]	71.1±24.1[38.7–151. 0]	48.6±15.7[21.9–75.2]	42.7±12.2[26.5–67.9]	<0.001 by design
**Height** (m)	1.58±0.13[1.32–1.83]	1.54±0.12[1.29–1.74]	1.58±0.20[1.14–1.88]	1.51±0.14[1.27–1.82]	0.8
**BMI-SDS**	2.3±0.4[1.3–2.9]	2.5±0.6[1.5–4.4]	0.1±0.7[−1.3–1.2]	−0.0±0.6[−1.2–1.0]	<0.001 by design
**Prepubertal/early pubertal** **(Tanner stages 1+2)** **(n; %)**	33 (73%)	22 (49%)	19 (56%)	16 (64%)	0.9 (comparison of early to late Tanner stages for normal weight vs. obese by chi-squared test)
**Pubertal/postpubertal** **(Tanner stages 3–5)** **(n; %)**	12 (27%)	23 (51%)	15 (44%)	9 (36%)	
**Serum blood glucose (0′)** **(mmol/l)**	4.95±0.47[3.42–6.02]		n.a.		
**Serum blood glucose** **(120’) (mmol/l)**	5.98±0.92[3.93–7.80]		n.a.		
**HOMA-IR**	3.35±1.84[0.87–14.38]		n.a.		
**Heart rate (bpm)**	79.3±11.8[45.0–118.4]		78.7±11.5[57.1–110.9]		n.s.
**Systolic blood** **pressures (mmHg)**	114.4±10.6[90.0–137.0]		n.a.		
**Diastolic blood** **pressures (mmHg)**	65.2±8.3[40.0–81.0]		n.a.		
**E/I-Ratio**	1.77±0.44[1.08–3.30]		1.95±0.37[1.40–2.78]		0.008
**ln(HF) (bpm2)**	2.00±0.88[−0.11–3.71]		2.32±0.70[0.57–3.85]		0.02
**ln(LF/HF)**	−0.32±0.71[−2.30–1.33]		−0.50±0.59[−2.09–0.93]		0.08
**PDD (mm)**	7.69±0.67[5.25–8.96]		7.94±0.60[6.30–9.24]		0.02
**RDV (mm/s)**	1.55±0.38[0.58–2.45		1.68±0.43[0.88–2.65]		0.07

Values are given as mean ± SD (first line) and min-max (except pubertal stages, second line). Group comparisons were performed by a two-sample t-test. (Missing values: E/I ratio: 1 measurement from an obese patient, RDV: 2 normal weight and 3 ov/ob patients).

### Procedures and Measurements

#### 1. Clinical and anthropometric data

A detailed medical history was obtained, and a physical examination was performed in all participants to exclude any concomitant disease. For anthropometric measurements, all children were assessed barefoot and only wearing light underwear. Body height was measured by the digital stadiometer “Dr. Keller III” (Günter GmbH, Tauscha, Germany; precision ±2 mm). Body weight was determined by a digital scale (SECA^®^-scale, Vogel & Halke GmbH, Hamburg, Germany; precision ±100 g). Body mass index (BMI) was calculated by the formula: weight in kilograms divided by the square of height in meters. BMI data were standardized to age and sex of the children applying German reference data [14] and were calculated as BMI-SDS according to the LMS method [15]. The BMI-SDS provides a normalized measurement for the degree of overweight or obesity. By applying the LMS method, it is assumed that BMI data of each age group are normally distributed [15]. A cut off ≥1.28 SDS (90^th^ centile) classifies overweight and a cut off ≥1.88 SDS (97^th^ centile) classifies obesity in children [14]. Pubertal stage was assessed according to Tanner stages [20].

In obese children, fasting plasma glucose, fasting insulin, transaminases (ALAT, ASAT, GGT), uric acid, triglycerides, cholesterol, HDL-cholesterol and LDL-cholesterol levels were measured by a certified laboratory. An oral glucose tolerance test (OGTT) was performed to exclude impaired glucose metabolism. For the OGTT, blood samples were collected at 0, 30, 60, 90, and 120 minutes after a glucose load of 1.75 g/kg body weight (maximum of 75 g glucose). Impaired glucose tolerance was defined as a glucose level >7.8 mmol/l 120 minutes after the glucose load [18]. To determine insulin sensitivity, the homeostasis model assessment for insulin resistance (HOMA-IR) was applied. HOMA-IR was calculated using the equation HOMA-IR = fasting insulin (µU/mL) x fasting glucose (mmol/l)/22.5 [21]. Blood pressure was obtained by the arithmetic mean of three single measurements by a certified device in the supine position after a rest period.

#### 2. Autonomic nervous function

For the characterisation of autonomic nervous function (sympathetic/parasympathetic activity), 3 different methods were applied. Measurements were performed in the afternoon between 1 and 3 pm, following a rest period of 15 minutes:

*2.1. Heart rate variability (HRV)*: To screen for cardiac autonomic function, HRV was measured in the resting position and following deep breathing via the computer-based system ProScicard (Medset Medizintechnik GmbH, Hamburg, Germany). Measurements were performed over a five minute period, and the time-domain and frequency-domain indices of HRV were analysed: root mean square of successive differences (RMSSD), power spectral analysis in the low frequency spectrum ln(LF); (0.05–0.15 Hz) and in the high frequency spectrum ln(HF); (0.15–0.5 Hz) and the low frequency/high frequency ratio (ln(LF/HF) ratio) were calculated. Measurements under deep breathing were performed over a period of 110 heart beats and a stable respiratory frequency of 6 cycles per minute. The ratio of the longest RR interval during expiration to the shortest interval during inspiration (E/I ratio) was calculated.

*2.2. Sympathetic skin response (SSR)*: For the assessment of sympathetic skin response, all children were investigated in a dark and silent room, in a supine position, with their eyes closed. Following a short rectangular electric stimulus (10 mA, 0.1 ms duration) over the glabella, sympathetic skin response was recorded with surface electrodes (palmar and plantar) with a dedicated device (Keypoint, Natus Europe GmbH, München, Germany). The mean latency of 2 responses, one from each side of the body, was calculated in order to screen for sympathic ANS dysfunction [6].

*2.3. Quantitative pupillography:* Using a pupillograph (AMTech Pupilknowlogy GmbH, Weinheim, Germany), pupil diameter in darkness (PDD) was assessed. Pupillary light reflex was measured over a period of 2 seconds (intensity of light stimulus: 10^4^ cd/m^2^, duration of stimulus 200 ms) and several parameters were obtained: relative light reflex amplitude, latency, constriction velocity, early re-dilation velocity (RDV). The mean value of 2 stable measurements of the left eye were used for further analyses [8].

### Statistics

The association between each of the dependent and independent variables was tested in a two stage process. In the first stage, the potential dependence of each variable on BMI-SDS, age, gender and pubertal stage was assessed in a linear model using Akaike’s information criterion [22]. Based on the assumption that height is likely to affect skin latency times, this variable was also included for those parameters. The model was simplified by dropping terms successively if their information contribution was negative, indicating that the association is at best very weak. In the second stage, the remaining terms in the final model were placed in decreasing order of information content and the lack-of-fit sum of squares of this nested-model structure was analysed with an F-test, a process equivalent to performing a likelihood ratio test. The significance level was placed at 5%, though a conservative Bonferroni-Holm adjustment [23] is also discussed for each of the complexes ‘heart rate variability’, ‘sympathetic skin response’ and ‘pupillography’. When a significant dependence on BMI-SDS was found, the multivariate analysis was augmented by including a two sample t-test between the obese and normal weight subjects with Welch approximation. Furthermore, Spearman’s rank correlation test was used to test whether or not those variables depend on HOMA-IR values. All analyses were performed using R version 2.11 [24].

## Results

A total of 154 children (mean age 12.0 years) were included in the study. Out of 95 obese children, 5 had to be excluded due to impaired glucose metabolism, which is in line with previously published prevalence rates of impaired glucose tolerance among children and adolescents with marked obesity [25]. Thus, data of 90 obese children (45 boys) and data of 59 normal weight children (34 boys) could be applied for further analyses (149 in total). Within the obese study group, 7 candidates (6.3%) presented with (slightly) elevated blood pressure measurements according to the recommendations of the American Heart Association [26], 17 candidates (15.3%) had elevated serum ASAT serum levels, 18 candidates (16.2%) had one pathological lipid measures (mainly decreased HDL-cholesterol), 5 candidates (4.5%) had 2 pathological lipid measures (mainly decreased HDL-cholesterol and elevated total cholesterol), none of the obese candidates had more than 3 pathological lipid findings, and 6 obese candidates presented with hyperuricaemia (5.4%). Some variables of interest had to be omitted from analyses due to the following reasons: light reflex amplitude: 1 value missing out of 154 (problems with measurement), early re-dilation velocity: 5 values missing out of 154 (problems with measurements), E/I ratio: 1 value missing out of 154 (problems with measurement), sympathetic skin response: 26 values missing out of 154 (measurement could not be performed on 6 normal-weight and 21 overweight/obese children due to missing informed consent of parents).

We did not find a significant association between any of the measured parameters and pubertal stage. The terms eliminated by the first stage of the analysis process will not be referred to explicitly below.

In order to see the concrete model of how the independent variables vary with the dependent ones, we have included the estimates for the coefficients in the linear model (Table 2).

**Table 2:** Coefficients for the significant covariates resulting from the linear models.

Dependent Variable	Independent Variables						
	Intercept	BMI-SDS	Age (years)	Gender (female = 1 male = 0)	Height (cm)	Gender × BMI-SDS (female = 1 male = 0)	Gender × age (years)
**E/I**	1.96 [1.86, 2.06]	−0.08 [−0.13, −0.03]	−	−	−	−	−
**ln(LF) (ln(bpm^2^))**	2.4 [1.8, 3.0]	−	−0.05 [−0.1, −0.0]	−	−	−	−
**ln(HF) (ln(bpm^2^))**	3.1 [2.5, 3.8]	−0.13 [−0.23, −0.02]	−0.076 [−0.13, −0.03]	0.28 [0.02, 0.53]	−	−	−
**ln(LF/HF)**	−0.51 [−0.68, −0.35]	0.08 [0.00, 0.17]	−	−	−	−	−
**Lower limb Latency (ms)**	712 [122, 1300]	−	−	−143 [−254, −32]	7.78 [4.07, 11.5]	−	−
**Upper limb Latency (ms)**	1460 [1400, 1520]	−	−	−95 [−180, −6]	−	−	−
**PDD (mm)**	8.2 [7.9, 8.4]	−0.2 [−0.3, −0.1]	−	−0.5 [−0.8, −0.1]		0.2 [0.0, 0.3]	−
**re-dilation velocity (mm/s)**	2.3 [1.9, 2.8]	−	−	−0.8 [−1.5, −0.2]	−	−	boys: −0.06 [−0.09, −0.02] girls: −0.00 [−0.03, 0.04]

Values for the coefficients in front of the independent variables are given together with a 95% CI. If a variable did not enter into a particular model, then this is indicated by a dash. The units in which the coefficients are measured can be inferred from those listed for the dependent and independent variables.

### Heart Rate Variability

**Figure 1:**
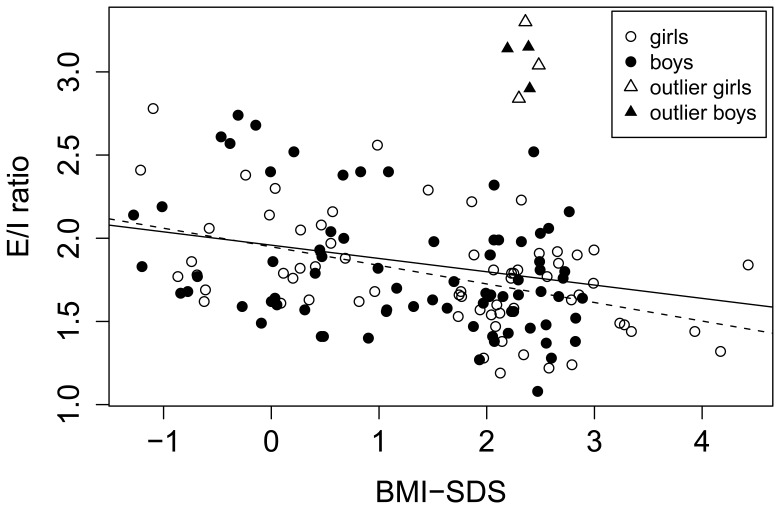
Correlation between E/I ratio (index of parasympathetic nervous system) and BMI-SDS in 149 normal-weight and obese children. The solid regression line is found using all the data points, whereas the dashed line is found upon eliminating the outliers marked as triangles. For all the data points, Pearson’s correlation coefficient = −0.24 (p = 0.003) and without the outliers, it is −0.41 (p = 3×10^−7^).

*E/I ratio* was negatively associated with BMI-SDS (p = 0.003) (Fig. 1). The dependence on age, used in the second stage of the analysis, was not significant (p = 0.1).

**Figure 2:**
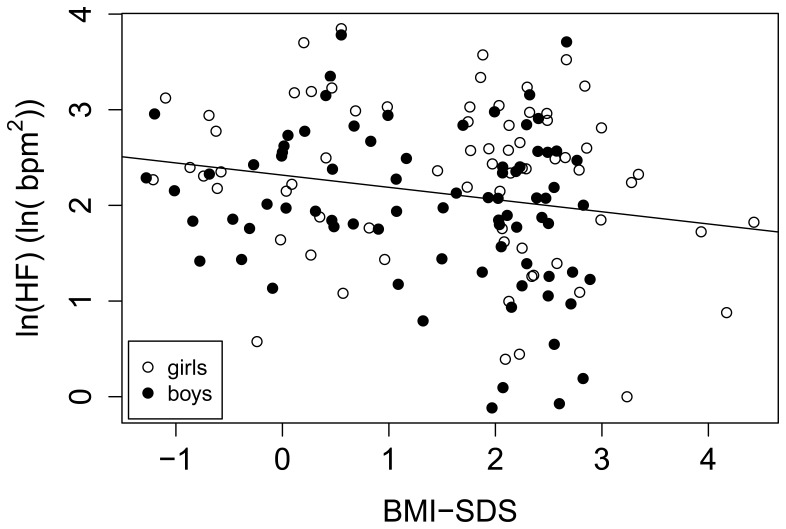
Correlation between ln(HF) (index of parasympathetic nervous system) and BMI-SDS in 149 normal-weight and obese children. Pearson’s correlation coefficient = −0.19, 95% CI = [−0.34, −0.03], (p = 0.03).

A two-sample t-test indicated that the mean E/I value was 0.18 lower for the obese group than for the normal weight peers (95% CI [0.05, 0.31]p = 0.008). Frequency domain measures for the logarithm of the *low frequency power (ln(LF)*) failed to yield a significant dependence on BMI-SDS, though age was found to be significantly associated (p = 0.03). Frequency domain measures for the logarithm of the *high frequency power (ln(HF)*), however, showed a significant relation to BMI-SDS, age, and gender All three variables explained a significant portion of the variance (p-values: 0.03 for BMI-SDS, <0.001 for age, 0.04 for gender). A two-sample t-test indicated that the mean for ln(HF) was 0.3 ln(bpm^2^) lower for obese than for the normal weight group (95% CI [0.06, 0.6] ln(bpm^2^), p = 0.02; Table 1). These results suggest decreased parasympathetic activity (Fig. 2). The logarithm of *ln(LF/HF)* showed a fairly weak, but significant positive dependence on BMI-SDS (p = 0.05). A two sample t-test showed a trend towards a difference between the obese and the normal weight group (p = 0.08; Table 1). The root mean square of successive differences (RMSSD) was only tested for its dependence on BMI-SDS, which was not found to be significant (p = 0.1) (Table S1).

### Sympathetic Skin Response

For analyses of sympathetic skin response, the parameters height and gender were retained in the second stage model for the upper and lower limbs and, furthermore, BMI-SDS was retained for the lower limbs. For the lower limbs, the linear model indicated a very strong positive dependence on height, as expected (p = 10^−5^), a significant dependence on gender (p = 0.01) and suggested some evidence for dependence on BMI-SDS (p = 0.09). As for the upper limbs, only gender was found to be significant (p = 0.04). There was not a significant dependence on height (p = 0.2). Group comparisons between the obese and normal weight participants are presented in Table S1.

### Quantitative Pupillography

**Figure 3:**
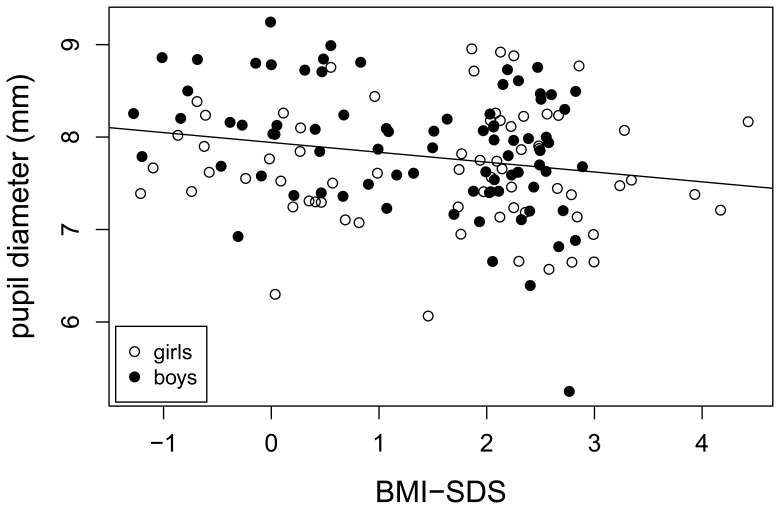
Correlation between pupil diameter (index of sympathetic nervous system) and BMI-SDS in 149 normal-weight and obese children. Pearson’s correlation coefficient = −0.21, 95% CI = [−0.36, −0.05], (p = 0.01).

**Figure 4:**
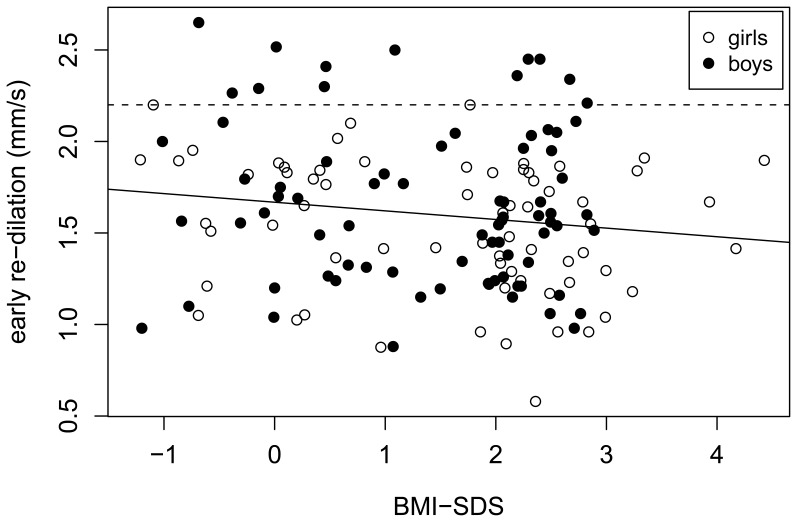
Correlation between early re-dilation (index of sympathetic nervous system) and BMI-SDS in 149 normal-weight and obese children. Pearson’s correlation coefficient = −0.15, 95% CI = [−0.30, 0.02], (p = 0.08). Note that only boys have values above 2.2 mm/s (dashed line). The average age of these boys was 11.1 and only one of them was older than 13. This helps explain the strong interaction between age and gender, i.e. that the age dependence for boys is significantly different than that for girls. The regression line showing the downward trend for increasing BMI-SDS is also shown (solid line).

There was a significant negative association between *pupil diameter in the dark (PDD)* and BMI-SDS (p = 0.01) (Fig. 3). Moreover, the terms gender (p = 0.06) and the interaction term BMI-SDS*gender (p = 0.03), i.e. that the dependence on BMI-SDS for boys is markedly different than that for girls, are borderline significant and significant, respectively. Comparing the obese with the normal weight group using a t-test showed that PDD was lower for the former by 0.25 mm (95% CI [0.04, 0.46]mm, p = 0.02). As for *early re-dilation velocity*, correlation to BMI-SDS (Pearson’s correlation coefficient = −0.15, 95% CI = [−0.30, 0.02]) suggests in trend a negative association (p = 0.08). Performing a t-test for BMI-SDS suggested that the obese group had a 0.13 mm/s slower re-dilation velocity than the normal weight peers (95% CI [−0.01,0.27]mm/s p = 0.07). In addition, the linear model took into account age, gender and BMI-SDS and demonstrated a significant contribution for the interaction term age×gender (p = 0.01), i.e. that the dependence on age for boys was markedly different from that for girls (Fig. 4).

Both, PDD and early re-dilation velocity suggest decreased sympathetic activity with increasing BMI-SDS. For *pupil latency,* the two-step analysis taking into account BMI-SDS, age, gender, and pubertal stage did not yield any significant dependencies (p = 0.07 for both pubertal stage and gender). For *relative amplitude and constriction velocity*, none of the measured parameters passed the first stage of the analysis. This lack of dependence is corroborated by F-tests in models taking any of them into account.

Additional results from comparing the obese with the normal weight group using a t-test are presented in Table S1.

### Dependence on HOMA-IR

A possible dependence on parameters of insulin resistance was analyzed by Spearman rank correlation. None of the four parameters that depended significantly on BMI-SDS was found to correlate significantly with HOMA-IR. The values for the correlation coefficients are −0.1, −0.1, 0.1 and −0.1 for E/I, ln(HF), ln(LF/HF) and PDD. The corresponding p-values are 0.6, 0.4, 0.2 and 0.2.

### Bonferroni-Holm Adjustment

After a conservative Bonferroni-Holm adjustment, the E/I ratio and PDD still depended significantly on BMI-SDS (adjusted p  = 0.01 and 0.05, respectively) whereas ln(HF), and ln(LF/HF) did not (adjusted p  = 0.1 for both measures).

## Discussion

The present study represents – to the best of our knowledge – the largest data set to date examining the influence of obesity on ANS function in the paediatric population. The findings reveal extensive abnormalities in obese children and adolescents. The distinct pattern of abnormalities provides a hint as to the pathogenesis of dysfunctional ANS in overweight and obesity.

### Strengths and Weaknesses in Relation to Other Studies

Obesity is known to be associated with disturbed ANS function in adults. Previous studies of heart rate variability assessments provided evidence for similar dysfunction in obese children [27]–[33]. In line with these previous observations [27]–[34]. we found diminished values for ln(HF) and E/I ratio with increasing BMI-SDS, suggesting decreased parasympathetic activity with increasing body weight. Although we did not assess physical activity in our subjects, decreased parasympathetic activity may indirectly rather than directly be related to obesity. A study in obese children matched for weight, but differing in the amount of physical activity demonstrated decreased parasympathetic activity in the physically inactive obese children relative to their physically active peers [35].

We found no significant interdependence between ln(LF) and BMI-SDS. Thus, analysis of heart rate variability did not provide evidence for decreased sympathetic activity in obese children. However, a weak positive dependence on BMI-SDS in ln(LF/HF) ratio was found. This finding is in line with several previous reports [28], [31], [36], but differs from others [30], [32], [37] in which ln(LF) increased with increasing BMI. One factor possibly explaining this discrepancy may be that abnormality of sympathetic cardiovascular autonomic function is related to the duration of obesity: A previous study examining heart rate variability revealed that parameters reflecting sympathetic activation were significantly increased in individuals with obesity of recent onset, but not in those with intermediate or long-standing obesity [38]. Although we did not record the duration of the obesity in our cohort, we consider it likely that most children have been obese for several years.

In addition to heart rate variability, ANS function testing in our study included sympathetic skin response and direct pupillography. These different methods also allow for assessment of both parasympathetic and sympathetic nervous function. SSR, PDD as well as re-dilation velocity are known to reflect sympathetic nervous system activity, whereas latency of pupillary constriction, constriction amplitude and velocity are markers of parasympathetic nervous system function [39]. Parameters of SSR for the lower and upper limbs provided little evidence for a relation to BMI-SDS, however without reaching statistical significance. This finding is in line with assessments in adults of sympathetic skin response revealing no differences between obese and normal weight subjects [7] and underlines that SSR is a rather insensitive test of ANS function.

Analyses of quantitative pupillography provided significantly negative associations between PDD and BMI-SDS and in trend a negative correlation between early re-dilation velocity and BMI-SDS, both suggesting decreased sympathetic activity with increasing body weight. Thus, pupillographic findings (especially that of PDD) provide strong and independent evidence for dysfunctional sympathetic activity function in obese children. Abnormalities may result from pathology at the level of the central processing pathway or the efferent sympathetic branches of the pupillary light reflex [8]. Pupillographic evidence of dysfunctional sympathetic activity function in obese children is noteworthy, because analysis of heart rate variability (see above) did not point to decreased sympathetic activity. Unlike direct pupillography [40], variables derived from HRV analysis [41]–[43] seem to be influenced by physical activity and exercise. Therefore, one might argue that normal sympathetic activity in HRV analyses could have been the result of physical training in our patient cohort. We consider this hypothesis unlikely because (i) obese patients were not enrolled in physical exercise programs at the time of the study and (ii) physical training would also have normalized cardiac parasympathetic nerve function, which was, however, abnormal. Therefore, pupillography seems to reveal abnormalities of sympathetic activity more readily than HRV analysis. In contrast to pupillographic evidence of sympathetic autonomic dysfunction, parameters reflecting parasympathetic activity in direct pupillography were not associated with BMI-SDS. In male adults, parasympathetic activity as assessed by direct pupillography decreased with increasing body fat content [44]. However, since the subjects in that study were of normal weight, the significance of normal parasympathetic activity in obese children remains unclear.

### Meaning of the Study

Our results suggest decreased parasympathetic and decreased sympathetic activity in obese children and adolescents that is not limited to one organ system. HVR and quantitative pupillography emerge as valuable methods to assess parasympathetic and sympathetic autonomic dysfunction in obese children and adolescents, whereas sympathetic skin response seems to lack sufficient sensitivity to detect ANS abnormalities.

If there is evidence of both parasympathetic and sympathetic nervous system dysfunction in obesity, then why did HRV analysis provide evidence only for parasympathetic autonomic dysfunction, and direct pupillography only for sympathetic autonomic dysfunction? Perhaps this conspicuous pattern of differential sensitivities can be understood best, when considering the differential anatomical pattern of autonomic innervations of both end-organs. The heart is innervated by long postganglionic parasympathetic fibres, but short postganglionic cardiac sympathetic fibres, whereas the pupil is innervated by long postganglionic sympathetic fibres but only short parasympathetic fibres. The pattern of abnormalities could be explained by assuming a length-dependent pathology, suggesting that the peripheral autonomic nerve function may be vulnerable in obesity. A peripheral, length-dependent autonomic neuropathy has previously been described in diabetic autonomic neuropathy [11], [44], [45]. Although by design all individuals were normoglycemic in the present study, the possibility must be considered that the pathogenesis of obesity-associated dysfunction of the peripheral ANS overlaps with that of diabetic autonomic neuropathy. Two previous studies found cardioautonomic parasympathetic dysfunction to be associated with insulin-resistance in obese children [46], [47]. In our study, HOMA-IR provided some evidence for insulin resistance in the adipose normoglycemic participants. However and importantly, none of the parameters that depended significantly on BMI-SDS was found to be significantly correlated with HOMA-IR. Therefore, dysfunctional peripheral ANS may develop via independent pathogenic pathways, a possibility deserving of further study.

### Strengths and Weaknesses of the Study

To the best of our knowledge, this work represents the first detailed comprehensive assessment of autonomic nervous system function in a large cohort of normal weight and obese, otherwise healthy, children. In particular the fact that only individuals with normal glucose metabolism were studied helped to rule out a major confounding factor. We did not assess duration of obesity and level of physical activity. As both factors might influence autonomic nervous function in adults or children with obesity [35], [38] this omission represents a limitation of the study.

### Unanswered Questions and Future Research

As data on autonomic nervous function is very limited to date, it remains unclear whether the autonomic dysfunction described herein represents a functional disturbance or the beginning of a neuropathy. The exact pathogenesis needs to be further elucidated. In addition, there are no data available to date, neither in adults nor in children, whether an obesity-induced neuropathy exists. It also remains unclear, whether the observed autonomic dysfunction in pediatric obesity might be reversible by weight loss or lifestyle intervention. Future studies should investigate the influence of physical activity or the duration of obesity on autonomic nervous system dysfunction in childhood obesity.

### Main Conclusions

Decreased parasympathetic and sympathetic activity is associated with increasing BMI-SDS in childhood and adolescence. This dysfunction may already involve several organ systems. The pattern of abnormalities suggests a length-dependent peripheral autonomic nervous system dysfunction. Obesity related autonomic nervous system dysfunction follows the same pattern as diabetic autonomic neuropathy. In addition to heart rate variability, quantitative pupillography may be useful in assessing the function of the ANS in children and adolescents.

## Supporting Information

Table S1 **Group comparison of additional autonomic nervous function measurements.** Values are given as mean ± SD (first line) and min-max (second line). Group comparisons were performed by a two-sample t-test.
(DOCX)

## References

[1] WHO (2011) Obesity and overweight. Fact sheet N°311. http://wwwwhoint/mediacentre/factsheets/fs311/en/indexhtml.

[2] Blüher S, Meigen C, Gausche R, Keller E, Pfaeffle R, et al (2011) Age-specific stabilization in obesity prevalence in German children: A cross-sectional study from 1999 to 2008. Int J Pediatr Obes 6: e199–206. 21091288 10.3109/17477166.2010.526305

[3] Han JC, Lawlor DA, Kimm SYS (2010) Childhood obesity. Lancet 375: 1737–1748. 20451244 10.1016/S0140-6736(10)60171-7 PMC3073855

[4] Beijers H, Ferreira I, Bravenboer B, Dekker J, Nijpels G, et al (2009) Microalbuminuria and cardiovascular autonomic dysfunction are independently associated with cardiovascular mortality: evidence for distinct pathways: the Hoorn Study. Diabetes Care 32: 1698–1703. 19564456 10.2337/dc08-1544 PMC2732136

[5] Arunodaya GR, Taly AB (1995) Sympathetic skin response: a decade later. J Neurol Sci 129: 81–89. 10.1016/0022-510x(94)00265-p 7608740

[6] Levy D, Reid G, Rowley D, Abraham R (1992) Quantitative measures of sympathetic skin response in diabetes: relation to sudomotor and neurological function. J Neurol Neurosurg Psychiatry 55: 902–908. 1331334 10.1136/jnnp.55.10.902 PMC1015186

[7] Priano L, Miscio G, Grugni G, Milano E, Baudo S, et al (2009) On the origin of sensory impairment and altered pain perception in Prader-Willi syndrome: a neurophysiological study. Eur J Pain 13: 829–835. 18986815 10.1016/j.ejpain.2008.09.011

[8] Heller P, Perry F, Jewett D, Levine J (1990) Autonomic components of the human pupillary light reflex. Invest Ophthalmol Vis Sci 31: 156–162. 2137115

[9] Pfeifer MA, Weinberg CR, Cook DL, Reenan A, Halter JB, et al (1984) Autonomic neural dysfunction in recently diagnosed diabetic subjects. Diabetes Care 7: 447–453. 6499637 10.2337/diacare.7.5.447

[10] Straub RH, Zietz B, Palitzsch KD, Scholmerich J (1996) Impact of disease duration on cardiovascular and pupillary autonomic nervous function in IDDM and NIDDM patients. Diabetes Care 19: 960–967. 8875089 10.2337/diacare.19.9.960

[11] Dütsch M, Marthol H, Michelson G, Neundörfer B, Hilz MJ (2004) Pupillography refines the diagnosis of diabetic autonomic neuropathy. J Neurol Sci 15: 75–81. 10.1016/j.jns.2004.04.008 15240199

[12] Karachaliou F, Karavanaki K, Greenwood R, Baum JD (1997) Consistency of pupillary abnormality in children and adolescents with diabetes. Diabet Med 14: 849–853. 9371477 10.1002/(SICI)1096-9136(199710)14:10<849::AID-DIA470>3.0.CO;2-W

[13] Karavanaki K, Davies AG, Hunt LP, Morgan MH, Baum JD (1994) Pupil size in diabetes. Arch Dis Child 71: 511–515. 7726610 10.1136/adc.71.6.511 PMC1030087

[14] Kromeyer-Hauschild K, Wabitsch M, Kunze D, Geller F, Geiß HC, et al (2001) Percentiles of body mass index in children and adolescents evaluated from different regional German cohorts. Monatsschr Kinderheilkd 149: 807–818.

[15] Cole TJ, Green PJ (1992) Smoothing reference centile curves: The lms method and penalized likelihood. Statistics in Medicine 11: 1305–1319. 1518992 10.1002/sim.4780111005

[16] Freeman R (2005) Autonomic peripheral neuropathy. Lancet 365: 1259–1270. 15811460 10.1016/S0140-6736(05)74815-7

[17] Smith A, Singleton J (2008) Impaired glucose tolerance and neuropathy. Neurologist 14: 23–29. 18195653 10.1097/NRL.0b013e31815a3956

[18] Weiss R, Dziura J, Burgert T, Tamborlane W, Taksali S, et al (2004) Obesity and the metabolic syndrome in children and adolescents. N Engl J Med 3: 2362–2374. 10.1056/NEJMoa031049 15175438

[19] O'Rahilly S, Farooqi SI (2005) The genetics of obesity in humans. Endotext com, chapter 8. Assessed at: http://www.endotext.org/obesity.

[20] Tanner J, Whitehouse R (1976) Clinical longitudinal standards for height, weight, height velocity, weight velocity, and stages of puberty. Arch Dis Child 51: 170–179. 952550 10.1136/adc.51.3.170 PMC1545912

[21] Matthews D, Hosker J, Rudenski A, Naylor B, Treacher D, et al (1985) Homeostasis model assessment: insulin resistance and beta-cell function from fasting plasma glucose and insulin concentrations in man. Diabetologia 28: 412–419. 3899825 10.1007/BF00280883

[22] Lambert E, Sari CI, Dawood T, Nguyen J, McGrane M, et al (2010) Sympathetic nervous system activity is associated with obesity-induced subclinical organ damage in young adults. Hypertension 56: 351–358. 20625075 10.1161/HYPERTENSIONAHA.110.155663

[23] Holm S (1979) A simple sequentially rejective multiple test procedure. Scandinavian Journal of Statistics 6: 65–70.

[24] Team RDC (2010) R: A language and environment for statistical computing. R Foundation for Statistical Computing, Vienna, Austria.

[25] Sinha R, Fisch G, Teague B, Tamborlane WV, Banyas B, et al (2002) Prevalence of impaired glucose tolerance among children and adolescents with marked obesity. N Engl J Med 346: 802–810. 11893791 10.1056/NEJMoa012578

[26] American Heart Association (2012) http://wwwheartorg/HEARTORG.

[27] Tonhajzerova I, Javorka M, Trunkvalterova Z, Chroma O, Javorkova J, et al (2008) Cardio-respiratory interaction and autonomic dysfunction in obesity. J Physiol Pharmacol 59 Suppl 6 709–718. 19218698

[28] Guizar JM, Ahuatzin R, Amador N, Sanchez G, Romer G (2005) Heart autonomic function in overweight adolescents. Indian Pediatr 42: 464–469. 15923693

[29] Gutin B, Barbeau P, Litaker MS, Ferguson M, Owens S (2000) Heart rate variability in obese children: relations to total body and visceral adiposity, and changes with physical training and detraining. Obes Res 8: 12–19. 10678254 10.1038/oby.2000.3

[30] Kaufman CL, Kaiser DR, Steinberger J, Kelly AS, Dengel DR (2007) Relationships of cardiac autonomic function with metabolic abnormalities in childhood obesity. Obesity (Silver Spring) 15: 1164–1171. 17495192 10.1038/oby.2007.619

[31] Martini G, Riva P, Rabbia F, Molini V, Ferrero GB, et al (2001) Heart rate variability in childhood obesity. Clin Auton Res 11: 87–91. 11570608 10.1007/BF02322051

[32] Nagai N, Matsumoto T, Kita H, Moritani T (2003) Autonomic nervous system activity and the state and development of obesity in Japanese school children. Obes Res 11: 25–32. 12529482 10.1038/oby.2003.6

[33] Vanderlei LC, Pastre CM, Freitas IF Jr, Godoy MF (2010) Geometric indexes of heart rate variability in obese and eutrophic children. Arq Bras Cardiol 95: 35–40. 20585738 10.1590/s0066-782x2010005000082

[34] Vanderlei LC, Pastre CM, Freitas Junior IF, Godoy MF (2010) Analysis of cardiac autonomic modulation in obese and eutrophic children. Clinics (Sao Paulo) 65: 789–792. 20835556 10.1590/S1807-5932201000080009 PMC2933128

[35] Nagai N, Moritani T (2004) Effect of physical activity on autonomic nervous system function in lean and obese children. Int J Obes Relat Metab Disord 28: 27–33. 14710167 10.1038/sj.ijo.0802470

[36] Paschoal MA, Trevizan PF, Scodeler NF (2009) Heart rate variability, blood lipids and physical capacity of obese and non-obese children. Arq Bras Cardiol 93: 239–246. 19851651 10.1590/s0066-782x2009000900007

[37] Soares-Miranda L, Alves AJ, Vale S, Aires L, Santos R, et al (2011) Central fat influences cardiac autonomic function in obese and overweight girls. Pediatr Cardiol 32: 924–928. 21638039 10.1007/s00246-011-0015-8

[38] Rabbia F, Silke B, Conterno A, Grosso T, De Vito B, et al (2003) Assessment of cardiac autonomic modulation during adolescent obesity. Obes Res 11: 541–548. 12690083 10.1038/oby.2003.76

[39] Electrophysiology TFotESoCatNASoPa (1996) Heart rate variability. Standard of measurements, physiological interpretation and clinical use. Circulation 93: 1043–1065. 8598068

[40] Karlsen RL, Soli N (1979) Changes in pupillary dynamics in young men during prolonged severe exercise. Acta Ophthalmol (Copenh) 57: 41–47. 419975 10.1111/j.1755-3768.1979.tb06657.x

[41] Lucini D, Milani RV, Costantino G, Lavie CJ, Porta A, et al (2002) Effects of cardiac rehabilitation and exercise training on autonomic regulation in patients with coronary artery disease. Am Heart J 143: 977–983. 12075252 10.1067/mhj.2002.123117

[42] Gutin B, Owens S, Slavens G, Riggs S, Treiber F (1997) Effect of physical training on heart-period variability in obese children. J Pediatr 130: 938–943. 9202616 10.1016/s0022-3476(97)70280-4

[43] Boutcher SH, Nugent FW, McLaren PF, Weltman AL (1998) Heart period variability of trained and untrained men at rest and during mental challenge. Psychophysiology 35: 16–22. 9499702

[44] Peterson HR, Rothschild M, Weinberg CR, Fell RD, McLeish KR, et al (1988) Body fat and the activity of the autonomic nervous system. N Engl J Med 318: 1077–1083. 3352710 10.1056/NEJM198804283181701

[45] Smith S, Smith S (1983) Evidence for a neuropathic aetiology in the small pupil of diabetes mellitus. Br J Ophthalmol 67: 89–93. 6821679 10.1136/bjo.67.2.89 PMC1039972

[46] Taşçılar M, Yokuşoğlu M, Boyraz M, Baysan O, Köz C, et al (2011) Cardiac autonomic functions in obese children. J Clin Res Pediatr Endocrinol 3: 60–64. 21750633 10.4274/jcrpe.v3i2.131 PMC3119442

[47] Rabbone I, Bobbio A, Rabbia F, Bertello M, Ignaccoldo M, et al (2009) Early cardiovascular autonomic dysfunction, beta cell function and insulin resistance in obese adolescents. Acta Biomed 80: 29–35. 19705617

