# Supplementary material for: Dysfunction of Autonomic Nervous System in Childhood Obesity: A Cross-Sectional Study
Source: PLoS One. 2013 Jan 24;8(1):e54546. doi: 10.1371/journal.pone.0054546 (PMC3554723; doi:10.1371/journal.pone.0054546)
Supplement: Table S1 — Group comparison of additional autonomic nervous function measurements. Values are given as mean ± SD (first line) and min-max (second line). Group comparisons were performed by a two-sample t-test. (DOCX) [file pone.0054546.s001.docx]

**Table S1.**

|  | **Ov/Ob Normal weight**  **(> 90^th^ percentile) (10^th^ – 90^th^ percentile)** | **p-value** |
| --- | --- | --- |

**ln(LF) (bpm2)** 1.69 ± 0.86 1.82 ± 0.67 0.3

**[**-0.73 – 3.52] **[**0.15 – 3.42]

**RMMSD** 55.4 ± 27.3 62.2 ± 27.9 0.1

**[**14.7 – 117.7] **[**19.2 – 143.4]

**SSR – lower limbs (ms)** 1825 ± 287 1905 ± 391 0.2

**[**990 - 2415] **[**1125 – 3435]

**SSR – upper limbs (ms)** 1374 ± 241 1463 ± 260 0.06

**[**650 - 1875] **[**730 - 2255]

**Light reflex relative amplitude** 22.6 ± 6.2 21.2 ± 6.3 0.2

**[**8.7 – 40.4] **[**8.7 – 39.2]

**Latency (s)** 0.247 ± 0.021 0.245 ± 0.021 0.6

**[**0.210 – 0.311] **[**0.212 – 0.297]

**Constriction velocity (mm/s)** 4.84 ± 0.98 4.72 ± 1.11 0.5

**[**2.16 – 7.40] **[**2.38 – 7.64]
